# An empirical study on job burnout among university counselors and the improvement of occupational happiness

**DOI:** 10.3389/fpsyg.2025.1471285

**Published:** 2025-02-17

**Authors:** Qing-Qing Liang, Fang Yin

**Affiliations:** ^1^School of Earth Science and Resources, Chang'an University, Xi'an, China; ^2^Shaanxi Key Laboratory of Land Consolidation, School of Land Engineering, Chang'an University, Xi'an, China

**Keywords:** ideological and political education, emotional intelligence, job burnout, empirical study, job satisfaction

## Abstract

University counselors undertake a significant amount of repetitive and trivial work. The high-pressure, high-load nature of their duties gradually erodes their enthusiasm and consumes considerable psychological resources, leading to a higher-than-average level of job burnout among university counselors. The article examines the practical work of university counselors, analyzing and validating the moderating role of emotional intelligence in the relationship between emotional labor and job burnout. In September 2023, a survey was conducted involving 520 university counselors from 25 different types of universities, located across six provinces in eastern, central, and western China. Using the General Information Questionnaire for University Teachers, the Emotional Labor Scale, the Emotional Intelligence Scale, and the Job Burnout Scale, this empirical study investigated job burnout and mental health among counselors. The study aimed to assess the level of job burnout among university counselors and to propose suggestions for enhancing their occupational happiness. The results indicate that burnout among university counselors increases with age, with counselors aged 26–30 experiencing the highest levels of depersonalization and burnout. Furthermore, it has been observed that emotional intelligence tends to escalate with advanced educational attainment. Counselors scored higher on the dimensions of deep acting and genuine emotional expression compared to surface acting. It is recommended to address burnout from both individual and organizational dimensions, with particular focus on counselors aged 26–30. Implementing targeted training programs to enhance emotional intelligence can help reduce job burnout. Efforts should be made to transform burnout into occupational attachment, achieving harmony between the individual and their profession.

## 1 Introduction

Job burnout, also known as “work burnout” or “occupational exhaustion” in some translations, is commonly regarded by researchers as a syndrome. There are three main manifestations: (1) Emotional Exhaustion: Also known as emotional depletion or burnout, this is the stress dimension of job burnout. It occurs when individuals' emotional and physical resources are over-exploited and not replenished in a timely and effective manner, leading to an imbalance and overdraft of emotional and physical resources. This results in employees exhibiting extreme fatigue symptoms such as irritability, anger, and fatigue, losing motivation at work, and it is the core dimension of job burnout. (2) Depersonalization: Also referred to as dehumanization or depersonalization, this belongs to the interpersonal dimension of burnout. When individuals invest too much emotional resources without timely and effective replenishment, they display indifference, avoidance, and negativity in their work. This manifests as coldness, detachment, avoidance, and cynicism toward work objects and colleagues. (3) Diminished Personal Accomplishment: Also known as low personal accomplishment, this pertains to the self-evaluation dimension of burnout. It is characterized by employees feeling useless in their work, lacking a sense of achievement, feeling incompetent, having low work efficiency, and perceiving themselves as worthless or experiencing a reduced sense of self-worth in their work (Shen, 2012; Huang and Wang, 2018; He et al., 2022). Research indicates that high emotional labor is often associated with the occurrence of job burnout (Coaston, 2017). This paper builds upon the existing research findings on the dimensions, measurement, and influencing factors of emotional intelligence, emotional labor, and job burnout (Brotheridge and Grandey, 2002; Tang et al., 2007; Wang, 2010). By integrating these findings with the practical experiences of university counselors, the study primarily investigates the influence of emotional labor and emotional intelligence on job burnout among counselors (Lazarus and Folkman, 1984). Furthermore, it analyzes and validates the moderating role of emotional intelligence in the relationship between emotional labor and job burnout among counselors (Mullen et al., 2017; Guest and Carlson, 2019). The research findings contribute to a better understanding of the current status of emotional intelligence, emotional labor, and job burnout among university counselors (Li, 2010; Wu, 2013; Huang, 2020). They can serve as a valuable reference for university management departments in developing practices related to emotional management and human resource allocation for counselors (Zhang et al., 2022). The article focuses on the group of university counselors in China, examines the practical work of university counselors, analyzing and validating the moderating role of emotional intelligence in the relationship between emotional labor and job burnout. In September 2023, a survey was conducted involving 520 university counselors from 25 different types of universities, including Chang'an University, located across six provinces in eastern, central, and western China: Shaanxi, Gansu, Henan, Wuhan, Guangxi, and Beijing. Using the General Information Questionnaire for University Teachers, the Emotional Labor Scale, the Emotional Intelligence Scale, and the Job Burnout Scale, The findings can assist university management departments in implementing effective intervention measures to alleviate job burnout among counselors, thereby reducing its severity (Acker, 2012; Smith et al., 2014; Tang, 2020). This can lead to increased occupational happiness, organizational performance, student satisfaction, and counselor job satisfaction.

## 2 Research hypothesis

### 2.1 The relationship between emotional labor and occupational burnout

The depletion of resources can lead to psychological stress and discomfort for individuals. If this resource depletion is not effectively replenished and supported, it may result in occupational burnout (Davis and Tuttle, 2017). We consider surface acting in emotional labor strategies as a form of disguised emotional expression. When individuals engage in surface acting, they need to suppress and conceal their true feelings. This requires effort and resources from the individual to regulate their emotions to meet the emotional requirements set by the organization (Mullen et al., 2019; Moore et al., 2023). Over time, this can lead to occupational burnout. Unlike surface acting, when individuals engage in deep acting, they strive to align their internal emotional experiences with the emotional rules required by the organization. This involves actively thinking, cognizing, and remembering to change their internal emotional experiences, resulting in a genuine emotional expression that meets the organizational requirements. Therefore, while individuals also need to exert effort and allocate resources to regulate emotions, “if deep acting is successful, it can bring positive effects such as self-satisfaction and self-fulfillment to employees” (Yang, 2015; Zhang and Yu, 2016). Consequently, individuals not only do not experience negative impacts, but there is also a possibility of reducing occupational burnout. When individuals engage in natural expression, their own emotions align closely with the emotions required by the enterprise or organization. Consequently, minimal effort is required, resulting in less emotional involvement. Both deep acting and natural expression can reduce emotional exhaustion (Song and Zhang, 2010; Shen, 2013; Lao, 2021). The natural and deep acting of college counselors can enhance job satisfaction. Based on these findings, the following hypothesis is proposed:

Hypothesis 1a: Surface acting in emotional labor positively predicts each dimension of occupational burnout.

Hypothesis 1b: Deep acting, as a component of emotional labor, is expected to exhibit a negative predictive relationship with each dimension of occupational burnout.

Hypothesis 1c: Authentic expression negatively predicts each dimension of occupational burnout.

### 2.2 The relationship between emotional intelligence and occupational burnout

Emotional intelligence is the ability of an individual to recognize and understand their own emotions as well as those of others, and to regulate and control their own emotions. Individuals with higher levels of emotional intelligence are better equipped to recognize and understand the reasons behind their negative emotions, allowing them to adjust these emotions more effectively and even mobilize positive emotions to facilitate task completion. People with high emotional intelligence typically possess a strong capacity for self-assessment of their emotions and for observing and evaluating the emotions of others. They also have a high ability to apply and control emotional regulation. It is reasonable to believe that individuals with high emotional intelligence experience lower levels of emotional exhaustion and job burnout. Numerous studies have demonstrated that “emotional intelligence has a significant negative effect on job burnout, with a particularly notable impact on the dimensions of depersonalization and reduced personal accomplishment in job burnout.” Reilly (1994) found in a study focusing on college counselors that emotional intelligence is significantly negatively correlated with job burnout. Liu (2021) conducted a sampling study on college teachers from three different types of schools, which indicated that the four dimensions of emotional intelligence of college teachers were negatively correlated with occupational burnout. Based on this, the following hypothesis is proposed:

Hypothesis 2: Each dimension of emotional intelligence has a negative predictive effect on each dimension of burnout.

### 2.3 The regulatory effect of emotional intelligence

College counselors interact with a large number of students and their families on a daily basis, requiring significant emotional labor. They address various professional or procedural queries from students and manage emotions effectively throughout this process (Zhang et al., 2017; Kim and Lambie, 2018). Therefore, good emotional expression, the ability to assess others' and one's own emotions, and emotional management skills are essential abilities and techniques in the work of college counselors. Individuals with high emotional intelligence can effectively manage and regulate their own emotions, thereby fostering “positive interpersonal relationships, and gaining support and recognition” (Zhang and Yu, 2015; Xu et al., 2015; Wang, 2020). Positive interpersonal relationships enable college counselors to more easily accomplish their tasks, reducing work-related stress (Akhtar and Khan, 2019). High emotional intelligence allows for the accurate and effective assessment of both others' and one's own emotions, utilizing emotional regulation mechanisms to create positive emotions and effectively address negative ones (Bardhoshi and Um, 2021; Zhang et al., 2022).

Based on existing research findings and reasoning, the following research hypothesis is proposed:

Hypothesis 3: Each dimension of emotional intelligence moderates the relationship between emotional labor and job burnout.

## 3 Research objectives and methodology

### 3.1 Research objectives and data collection method

The survey respondents are university counselors from 25 different types of universities located in the eastern, central, and western regions of China. These universities include Chang'an University, Lanzhou University, Zhengzhou University, Wuhan University, Northwestern Polytechnical University, Guangxi University, Xi'an University of Science and Technology, Nanning Vocational and Technical College, China University of Geosciences (Beijing), among others, from six provinces, municipalities, or autonomous regions, namely Shaanxi, Gansu, Henan, Hubei, Guangxi, and Beijing. They must meet the following criteria:

(1) Have been employed for at least 1 year. (2) Voluntarily participate in the survey for this research project. A cross-sectional survey method was employed. With the assistance of the personnel departments and relevant colleges at each university, questionnaires were distributed to counselors. The counselors were provided with instructions on how to fill out the questionnaires. The surveyors collected the completed questionnaires within 7 days.

This survey, conducted in September 2023, distributed 520 questionnaires, of which 488 were returned as valid responses. The effective response rate was 93.85%. Please refer to Table 1 for details.

**Table 1:** Basic demographic statistics of 488 counselors from 25 universities in six provinces.

**Indicator**	**Group category**	**Number**	**Composition ratio (%)**	**Indicator**	**Group category**	**Number**	**Composition ratio (%)**
Age (years)	21 to < 26	152	31.15	Departmental grouping	Science and engineering	316	64.75
	26 to < 31	178	36.48		Liberal arts	112	22.95
	31 to < 36	91	18.65		Other	60	12.3
	≥36	67	13.73	Years of work experience (a)	0 to < 6	259	53.07
Gender	Male	6	1.23		≥6	229	46.72
	Female	482	98.77	Number of team members led grouping	0 to < 60⋏	249	51.02
Marital status	Unmarried	207	42.42		≥60⋏	134	27.46
	Married	275	56.35	Monthly income grouping	< 7,000	316	64.75
Education level	Other	6	1.2		≥7,000	157	32.17
	Bachelor's degree	22	4.51	Title grouping	Lecturer	249	51.02
	Master's degree	331	67.83		Associate Professor	215	44.06
	Doctorate	133	27.25		Professor	23	4.71
Overtime	Primarily on weekdays	274	56.15				
	Primarily on weekends	184	37.75				

⋏Denote the common elements between two sets.

### 3.2 Research methodology

This research employs an empirical approach based on questionnaire surveys. The survey instrument utilized is a scale with high reliability and validity from Western sources, which have undergone corresponding reliability and validity studies within Chinese populations and have been demonstrated to be suitable for use among Chinese populations (Zhang et al., 2017; Tan et al., 2022).

#### 3.2.1 Research proposal

**Figure 1:**
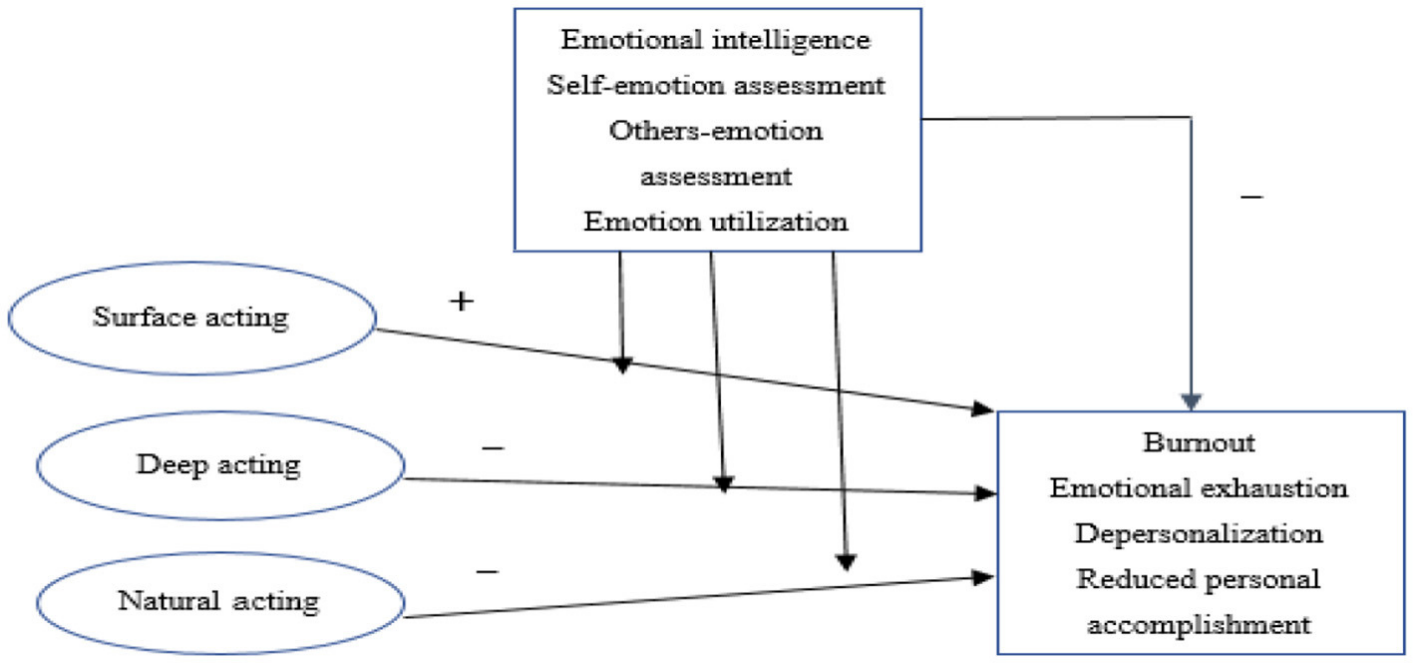
Preliminary hypothetical model.

Based on the aforementioned analysis and assumptions, this study aims to investigate the impact of emotional labor strategies on job burnout, the impact of emotional intelligence on job burnout, and the moderating effect of emotional intelligence on the relationship between emotional labor and job burnout. A preliminary hypothetical model has been established, as seen in Figure 1.

#### 3.2.2 Measurement tools

##### (1) Survey form for university teachers general information

Contents include: Age, Gender, Educational Background, Department, Job Title, Years of Work Experience, Overtime Situation, Average Monthly Income, Marital Status, Academic Qualifications.

##### (2) Emotional Labor Scale

The Emotional Labor Strategy Questionnaire mainly includes three dimensions of surface acting, deep acting, and natural expression, totaling 14 questions. It is primarily used to measure the actual situation of emotional labor among counselors. We adopted a Six-point Likert scale to evaluate the items. Scores from 1 to 6 represent Never, Rarely, Occasionally, Sometimes, Often, Always, respectively. A higher score indicates that counselors use the strategy more frequently.

##### (3) Emotional Intelligence Scale

The Chinese version of the Emotional Intelligence Scale (WLEIS-C) was established based on the Wong and Law Emotional Intelligence Scale (WLEIS), translated and revised by Wang Yefei. We utilized a Seven-point scale to evaluate the items, ranging from 0 to 6, representing Strongly Disagree, Somewhat Disagree, Disagree, Neutral, Somewhat Agree, Agree, and Strongly Agree, respectively. A higher score indicates higher emotional intelligence.

##### (4) Occupational Burnout Scale

The scale utilized is the Chinese version of the Occupational Burnout Inventory developed by Chinese scholars, tailored for the cultural context of China. We employed a Four-point scale to evaluate the items, ranging from 1 to 4, representing “Strongly Disagree,” “Disagree,” “Agree,” and “Strongly Agree,” respectively. A higher score indicates more severe burnout.

#### 3.2.3 Statistical analysis techniques

Valid questionnaires were inputted into Epidata software, and data entry was cross-checked to ensure accuracy. Statistical analysis was conducted using SPSS 19.0 software, with a significance level set at *P* ≤ 0.05, indicating statistically significant differences. All *P*-values are two-tailed probabilities. In this regard:

(1) Independent sample *t*-tests and one-way ANOVA were conducted on demographic data grouped differently with Emotional Intelligence, Emotional Labor Scale, and dimensions of Nursing Occupational Burnout, respectively. (2) Pearson correlation coefficient was used to examine the relationship between demographic data and Emotional Intelligence, Emotional Labor Scale, and dimensions of Nursing Occupational Burnout, aiming to understand if significant correlations exist. This laid the foundation for regression analysis. (3) Pearson correlation coefficient was employed to examine the relationships between dimensions within each scale and between dimensions of Nursing Occupational Burnout, aiming to understand if significant correlations exist. This laid the foundation for regression analysis. (4) The results indicate that, in terms of age distribution, individuals aged < 31 years accounted for 67.62% of the total sample, while those aged ≥31 years accounted for 32.38%, indicating a relatively young population. In terms of educational background distribution, individuals with a master's or doctoral degree accounted for 95.08% of the total sample. This indicates that the surveyed population generally has a high level of education, ensuring accurate understanding of the questionnaire items.

## 4 Research findings

### 4.1 Reliability test

As both the Emotional Labor Scale and the Emotional Intelligence Scale are mature instruments, reliability testing was not conducted in this study. The University Teachers' Occupational Burnout Scale, validated by Zhang (2015) among university teachers, has demonstrated good reliability and validity, indicating its suitability for counselors in China.

### 4.2 Scores of each dimension of the scales

Statistical results indicate that: (1) the scores for the Deep Acting dimension (4.62 ± 0.73) and the Natural Expression dimension (4.37 ± 0.96) were higher than the scores for the Surface Acting dimension (3.11 ± 1.05), as shown in Table 2. The results indicate that counselors commonly employ deep acting as their primary mode of interaction when facing students, followed by natural expression. Surface acting, however, is relatively less frequently used in these interactions. (2) The mean scores for the dimension of assessing others' emotions are relatively small, indicating that counselors pay less attention to students' emotional changes in their work. This warrants attention from university administrators. (3) Among the dimensions of counselor burnout, the mean score for emotional exhaustion is the highest at 2.60, followed by reduced personal accomplishment, and depersonalization has the lowest mean score. The total burnout score is 2.30.

**Table 2:** Scores of various dimensions of scales among 488 counselors from 25 universities in six provinces.

**Scale**	**Item**	**Score**	**Scale**	**Dimension**	**Score**
Emotional labor	Surface acting	3.11 ± 1.05		Emotion management	3.88 ± 1.03
	Deep acting	4.62 ± 0.73		Total emotional intelligence score	4.11 ± 0.86
	Natural expression	4.37 ± 0.96	Occupational burnout	Emotional exhaustion	2.61 ± 0.74
	Total emotional labor score	4.10 ± 0.45		Depersonalization	2.11 ± 0.61
Emotional intelligence	Self-emotion appraisal	4.41 ± 0.94		Reduced personal accomplishment	2.19 ± 0.70
	Others' emotion appraisal	4.01 ± 0.90		Total burnout score	2.30 ± 0.60
	Emotion regulation	4.18 ± 0.99			

#### 4.2.1 Scores of various scale dimensions among different age groups

Statistical analysis reveals the following insights: (1) Younger counselors utilize surface acting less than older counselors, with a statistically significant difference between age groups (*P* < 0.05). Conversely, older counselors employ deep acting more than younger counselors, with a statistically significant difference between age groups (*P* < 0.05). Moreover, the overall emotional labor burden is higher among older counselors compared to younger counselors. (2) Emotional Intelligence Scale and Emotion Management Dimension for Senior Counselors. Counselors in different age groups scored higher as their age increased, and the test results indicated that the differences were statistically significant (*P* < 0.01). (3) The data in the table also show that depersonalization decreases after initially increasing with age, with the 26–30 age group having the highest depersonalization score of 2.24. Burnout dimension shows a decreasing trend after initially increasing with age, with the 26–30 age group having the highest burnout score of 2.42. Analysis of variance (ANOVA) for different age groups indicates that the differences in depersonalization and burnout scores are statistically significant (both *P* > 0.05). See Table 3 for details.

**Table 3:** Scores of different dimensions for counselors of various age groups from 25 universities in six provinces (*N* = 488).

**Dimensions**	**Age (years)**	**Number of people**	**Score (*x* ±*s*)**	***F*-value**	***P*-value**
Emotional labor	21 to < 26	152	3.64 ± 0.03	0.01	0.06
	26 to < 31	178	4.04 ± 0.44		
	31 to < 36	91	4.09 ± 0.45		
	≥36	67	4.22 ± 0.43		
Emotional intelligence	21–25	152	4.08 ± 0.81	1.79	0.15
	26–30	178	4.04 ± 0.88		
	31–35	91	4.22 ± 0.77		
	≥36	67	4.28 ± 0.99		
Occupational burnout	21 to < 26	152	2.23 ± 0.64	2.76	0.04
	26 to < 31	178	2.42 ± 0.53		
	31 to < 36	91	2.33 ± 0.62		
	≥36	67	2.27 ± 0.65		

#### 4.2.2 Analysis of differences in various dimensions by educational background, marital status, and years of service

The score for evaluating others' emotions in the group with a master's degree or above was (4.10 ± 0.45), which showed a statistically significant difference compared to the group with a bachelor's degree or below according to the independent samples *t*-test (*P* < 0.05). The average score for emotional application in the group with a master's degree or above was 4.43, and this also showed a statistically significant difference compared to the group with a bachelor's degree or below (*P* < 0.01). The average emotional intelligence score for the group with a master's degree or above was 4.25, which showed a statistically significant difference compared to the group with a bachelor's degree or below according to the independent samples *t*-test (*P* < 0.05). Emotional intelligence increases with higher educational levels. The scores for emotional labor, emotional intelligence, and burnout dimensions across different marital status groups showed no statistically significant differences (all *P* > 0.05).

#### 4.2.3 Analysis of differences in various dimensions and total scale scores by department and number of supervised individuals

There are differences in occupational burnout among departments. As shown in the table, scores for emotional exhaustion, reduced personal accomplishment, and overall burnout gradually increase across the Science and Engineering, Humanities, and other departments. Analysis of variance confirms that these score differences are statistically significant (*P* < 0.01). The difference in depersonalization dimension scores was found to be statistically insignificant through variance analysis (*P* > 0.05; see Table 4. Several researchers have reached the same conclusion in their studies. Liberal arts advisors generally need to invest more emotional labor in their daily work, and they also face heavier workloads due to a relatively lower number of advisors compared to those in science and engineering.

**Table 4:** Scores of various dimensions for counselors from 25 universities in six provinces, differentiated by department and number of supervised individuals (*N* = 488, *x* ±*s*).

**Item**	**Number of individuals**	**Emotional exhaustion**	**Depersonalization**	**Reduced personal accomplishment**	**Occupational burnout**
**Department**					
Science and engineering	316	2.54 ± 0.70	2.13 ± 0.61	2.13 ± 0.66	2.27 ± 0.58
Liberal arts	112	2.72 ± 0.75	2.09 ± 0.55	2.32 ± 0.74	2.38 ± 0.58
Other	60	2.86 ± 0.87	2.3 ± 0.68	2.39 ± 0.80	2.52 ± 0.71
*F*-value		6.1	2.54	5.79	5.09
*P*-value		0.01	0.08	0.01	0.01
Number of supervised students	0 to < 60	2.54 ± 0.71	2.08 ± 0.61	2.12 ± 0.66	2.25 ± 0.58
	≥60	2.76 ± 0.78	2.27 ± 0.56	2.33 ± 0.77	2.45 ± 0.63
*t*-value		−2.78	−2.95	−2.76	−3.18
*P*-value		0.01	0.01	0.01	0.01

Additionally, as shown in Table 4, the burnout scale scores for “emotional exhaustion,” “depersonalization,” and “reduced personal accomplishment” are higher in the group with over 60 students compared to other groups. The differences in scores were found to be statistically significant through independent sample *t*-tests (*P* < 0.01). It also shows that advisors with a higher number of students experience greater levels of emotional exhaustion, depersonalization, and reduced personal accomplishment compared to those with fewer students. Advisors with more students have to deal with a greater variety of situations and emotions, requiring them to invest more emotional labor. Additionally, their overall workload is higher compared to advisors with fewer students, making them more prone to burnout.

## 5 Conclusions

### 5.1 The moderating role of emotional intelligence on emotional labor and job burnout

The statistical analysis reveals substantial correlations among the various facets of emotional labor and job burnout. This raises the question: How does emotional labor contribute to job burnout, and what is the predictive power of emotional intelligence concerning burnout? Additionally, what is the mediating role of emotional intelligence between emotional labor and burnout? Concentrating on the “emotional exhaustion” component of burnout, it is evident that, after accounting for demographic factors, surface acting in emotional labor positively forecasts “emotional exhaustion,” whereas deep acting has a significant negative predictive effect on “emotional exhaustion.” Furthermore, the “emotion management” aspect of emotional intelligence negatively predicts “emotional exhaustion.”

When controlling for the effects of the three dimensions of emotional labor and the four dimensions of emotional intelligence on “emotional exhaustion,” the inclusion of the interaction term SEA^*^SA in the model yields a significant Beta value, suggesting that the interplay between “self-emotion appraisal” and “surface acting” significantly influences “emotional exhaustion.” This indicates that “self-emotion appraisal” acts as a moderator in the relationship between “surface acting” and “emotional exhaustion,” with the implication that as the capacity for “self-emotion appraisal” increases, the influence of “surface acting” on “emotional exhaustion” diminishes.

This revised version aims to enhance clarity and flow while maintaining the original meaning of the text. It provides a more polished and academic tone suitable for scholarly writing.

The research findings highlight that the Beta value for the interaction term SEA^*^NA is statistically significant, implying that the interplay between self-emotion appraisal and natural acting exerts a substantial influence on “emotional exhaustion.” However, for a variable to act as a moderator, it is crucial that the independent variable has a significant effect on the dependent variable. This study's outcomes demonstrate that “natural acting” does not exert a significant predictive influence on “emotional exhaustion,” thus invalidating the moderating relationship between “self-emotion appraisal” and “natural acting.”

Upon adjusting for demographic variables, it is observed that surface acting within emotional labor positively forecasts “depersonalization” in burnout, whereas deep acting significantly and negatively predicts “emotional exhaustion.” Moreover, “natural acting” is also found to significantly and negatively predict “emotional exhaustion.” Furthermore, the “emotion regulation” component of emotional intelligence is identified as a significant negative predictor of “depersonalization.”

This refined passage aims to clarify the statistical relationships and the conditions for moderation, while also providing a more structured presentation of the study's outcomes. After controlling for demographic variables, surface acting in emotional labor positively predicts “reduced personal accomplishment” in burnout. Emotional intelligence's “emotion regulation” significantly negatively predicts “reduced personal accomplishment.” After controlling for the effects of the three dimensions of emotional labor and the four dimensions of emotional intelligence on “reduced personal accomplishment,” the Beta value of the interaction variable ROE^*^NA becomes significant when the interaction variable is included in the equation. However, the premise for moderation is that the independent variable significantly influences the dependent variable. The results of this study indicate that “natural acting” does not have a significant predictive effect on “reduced personal accomplishment.” Therefore, the moderation relationship between “emotion regulation” and “natural acting” does not hold.

The moderating effect of emotional intelligence on the relationship between emotional labor and burnout, as delineated by Zhang et al. (2022), is characterized by the following observations:

(1) Surface acting is positively associated with “emotional exhaustion,” “depersonalization,” and “reduced personal accomplishment.” Conversely, deep acting is negatively linked to “emotional exhaustion” and “depersonalization.”
(2) “Emotion regulation” within emotional intelligence is negatively predictive of “emotional exhaustion.” Similarly, “emotion utilization” is negatively correlated with “depersonalization” and “reduced personal accomplishment.”
(3) “Self-emotion appraisal” acts as a moderator in the relationship between “surface acting” and “emotional exhaustion,” while “emotion regulation” moderates the link between “surface acting” and “depersonalization.”

Drawing from these research findings, a clear picture emerges of how, after accounting for demographic variables, emotional intelligence and the interaction terms of emotional labor, when incrementally included in the regression model, illustrate the intricate impact relationships among the dimensions of emotional labor, emotional intelligence, and the various components of job burnout.

### 5.2 Direct impact of emotional labor strategies on occupational burnout

The research findings indicate that surface acting by counselors has a positive and significant influence on all three dimensions of burnout: “emotional exhaustion,” “depersonalization,” and “reduced personal accomplishment.” Previous studies have consistently shown a positive correlation between surface acting and emotional exhaustion, depersonalization, and reduced personal accomplishment. This reaffirms that surface acting serves as an effective predictive factor for emotional exhaustion and depersonalization. This pretense and suppression of emotions can lead counselors to perceive their work as meaningless and contrary to their own desires. Moreover, this pretense of emotions can mask the expression of counselors' natural behaviors, making individuals feel as though they are merely disguising themselves to fulfill professional images and ethics. The lack of fulfillment in meeting psychological needs at work, coupled with the absence of personal achievement satisfaction, contributes significantly to emotional exhaustion and depersonalization among counselors. Deep acting behavior is found to have a significant negative predictive effect on emotional exhaustion and depersonalization. Some studies have found that “deep acting” can positively predict reduced personal accomplishment. However, deep acting does not significantly predict emotional exhaustion or depersonalization (Huang and Wang, 2018; He et al., 2022).

The study suggests that counselors who employ deep acting are adept at utilizing emotion utilization and emotion regulation to effectively alter their inner feelings. Consequently, they project positive, friendly, and uplifting emotions when interacting with students, families, and colleagues. This ability contributes to fostering strong interpersonal relationships and experiencing job satisfaction. Individuals with a sense of accomplishment in their work tend to have a pleasant emotional state and are less likely to experience emotional exhaustion and depersonalization (Hochschild, 1983; Maslach et al., 2001).

Counselors' “natural behavior” has a significant negative predictive effect on “depersonalization.” Counselors who demonstrate care and compassion toward students in their work foster positive teacher-student relationships, receive higher evaluations from students (Ahmed et al., 2020), and achieve a higher quality of ideological and political education. Counselors with high-quality ideological and political education are more likely to garner recognition and support from colleagues and students, thereby fostering positive interpersonal relationships. Positive interpersonal relationships encourage counselors to actively engage with students, leading to more personalized communication. This reduces the likelihood of depersonalization or disintegration of personality among counselors.

The results indicate that there is a statistically significant difference between the scores of individuals with a master's degree or higher and those with a bachelor's degree or lower in the dimensions of “other emotion appraisal,” “emotion utilization,” and the total score of emotional intelligence, suggesting a correlation between emotional intelligence and educational level. Emotional intelligence increases as educational level rises. The study suggests that there is a correlation between emotional intelligence and education. Counselors with a master's degree or higher have undergone more extensive education and training, thereby possessing a greater theoretical understanding of emotional accumulation and placing greater emphasis on emotional experiences compared to counselors with a bachelor's degree or lower.

At the same time, the results demonstrate that individuals in different tenure groups score significantly differently on various dimensions of WLEIS-C (Parmar et al., 2022), as confirmed by statistical analysis. Emotional intelligence tends to increase with age and experience. From this table, it can be observed that individuals with different lengths of tenure score higher when they have longer tenure compared to those with shorter tenure. The scores of tenure groups on WLEIS-C compared to age in various dimensions of the scale show significant differences across more dimensions as tenure increases. This could be attributed to the continuous accumulation of experience in practical work, leading counselors to gain a deeper understanding of students and families, accumulate emotional experience, and become increasingly adept at controlling their emotions. Consequently, emotional intelligence gradually increases.

### 5.3 The direct impact of emotional intelligence on occupational burnout is evident

The study findings reveal a positive correlation between age and the overall emotional labor burden, indicating that older counselors tend to have more advanced and comprehensive strategies for handling emotional labor. This suggests that with age comes a richer tapestry of experience in the expression of emotional labor. Older counselors are more adept at articulating their emotional labor strategies, and they are also more inclined to utilize emotional expression tactics within their emotional labor repertoire. It can also be observed that older counselors have higher emotional intelligence scores compared to younger counselors (Zhang and Yu, 2016). Research abroad has shown that emotional intelligence increases with age and experience, with age being positively correlated with emotional intelligence (Mayer and Salovey, 1997; Brotheridge and Lee, 2002). Domestic researchers have also reached similar conclusions. As counselors age, they show improvements in self and others' emotional appraisal as well as in emotional regulation and utilization, indicating that counselors enhance their emotional intelligence through the trials and experiences of work and life events. This also suggests that emotional intelligence can be enhanced through training. In terms of counselor burnout, depersonalization and overall burnout scores show an initial increase with age followed by a decline. The age group of 26–30 years exhibits the highest scores in depersonalization and overall burnout. There are statistically significant differences in scores for depersonalization and burnout among different age groups. This age group of counselors faces higher levels of stress compared to other age groups. Many counselors in this age group have been involved in ideological and political education for 6–10 years. Perhaps they are experiencing the “7-year itch” of their careers, facing pressures related to job promotion, family responsibilities, and high expectations from students and family members in their work. These factors may be wearing down their passion for this profession. As counselors enter the subsequent age group, their emotional intelligence tends to increase while their work experience becomes richer. They become more familiar with their colleagues in the workplace, and their strategies for managing emotional labor become more skillful and adept. Consequently, burnout begins to decline.

The statistical findings underscore a robust negative correlation between the scores of the four dimensions of emotional intelligence among counselors and the three dimensions of counselor burnout. Hierarchical linear regression analysis further demonstrates that “emotion management” negatively predicts “emotional exhaustion,” while “emotion utilization” negatively predicts “depersonalization” and “reduced personal accomplishment.” That is, counselors with stronger emotional management skills or emotional utilization abilities experience lower levels of occupational burnout (He et al., 2022).

### 5.4 Implications for counseling practice

#### 5.4.1 Individual aspect

Counselors shoulder a significant amount of repetitive and mundane tasks in their work. The high-pressure and high-load nature of their work is also eroding their enthusiasm, depleting their psychological resources, leading to a higher level of occupational burnout among counselors compared to the norm. Through this study, counselors can reduce the level of occupational burnout and enhance their professional attachment and job satisfaction by focusing on the following two points:

##### 1. By enhancing their own emotional intelligence, counselors can reduce the occurrence of occupational burnout and increase professional attachment.

Research indicates that improving counselors' emotional utilization and emotional management both decrease the level of occupational burnout. Counselors should focus on enhancing their emotional intelligence in their work. As counselors gain more experience, their emotional intelligence tends to increase. Practical work experience serves as a beneficial pathway for enhancing counselors' emotional intelligence. Counselors should pay attention to improving their ability to regulate and control emotions in their work to avoid the psychological energy drain caused by emotional conflicts, thereby reducing the level of occupational burnout. In addition, counselors can enhance their emotional management and control abilities and reduce the occurrence of burnout by attending relevant training courses.

##### 2. During the process of emotional labor, counselors should minimize the use of surface acting strategies and instead rely more on deep acting and natural behavior. This can help enhance their job satisfaction.

When facing students who are experiencing various difficulties such as academic or psychological challenges in their work, counselors should not simply feign sympathy temporarily. Instead, they should utilize cognitive regulation techniques to internalize the required professional emotions as their own natural emotions. Through techniques like scenario simulation, counselors can evoke empathetic understanding and care, enabling genuine emotional connection with students. This approach helps reduce the need for pretense in facial expressions and body language, thereby decreasing the consumption of psychological energy. As a result, it can lower the level of occupational burnout and enhance job satisfaction.

#### 5.4.2 Organizational aspect

##### 1. Counselors with larger caseloads are more prone to experiencing burnout. Additionally, counselors predominantly involved in liberal arts disciplines exhibit significantly higher levels of burnout compared to those in science and engineering disciplines.

The school's management department should take into account the appropriate number of students assigned to counselors when planning counselor allocations. Additionally, they can consider implementing a rotation system for counselors among different departments to prevent counselors in departments with higher burnout levels from experiencing prolonged burnout, which could lead to adverse psychological and physiological effects, or even resignation.

##### 2. Counselor burnout increases with age, with counselors aged 26–30 exhibiting the highest levels of depersonalization and burnout.

The school's management department should pay special attention to counselors in this age group and provide timely counseling and stress relief to prevent experienced counselors in this age group from resigning due to unresolved occupational burnout. This could lead to counselor turnover, which would impact the development of the ideological and political education talent pool and even hinder the establishment of management talent teams.

##### 3. Emotional intelligence of counselors tends to increase with age, and counselors with weaker emotional management and utilization abilities are more prone to experiencing tendencies toward burnout.

The school's management department can enhance counselors' emotional intelligence abilities by offering relevant training courses. These courses can focus on improving counselors' emotional appraisal, emotion utilization, and control abilities, enabling them to adeptly handle various conflicts and contradictions in their work.

##### 4. Counselors with high emotional intelligence can mitigate the impact of emotional labor on burnout through regulatory mechanisms. They are better equipped to cope with occupational burnout and understand how to explore pathways to enhance job satisfaction.

Therefore, the school's management department can consider evaluating the emotional intelligence of candidates during the recruitment process to hire suitable counselors. Additionally, they can consider implementing relevant courses to enhance teachers' emotional intelligence, job satisfaction, and professional attachment.

## 6 Study limitations and future research counseling implications

Due to limitations in time, energy, and the researcher's capabilities, this study, despite yielding some valuable results, has several shortcomings:

(1) Sample Selection: The study utilized 488 valid questionnaires, which meets the sample size requirements for survey research. However, the use of a sampling survey means the subjects may not be broadly representative. As a result, it remains uncertain whether the findings are generalizable and applicable to a wider context.
(2) Research Methods and Tools: This study employed a cross-sectional questionnaire survey, a relatively straightforward method. Moreover, the scales and instruments used are not widely adopted domestically. The wording of the items may not fully adhere to the conventions of Chinese expression. Additionally, due to the author's limited expertise, exploratory and confirmatory factor analyses of the scales were not conducted, which are necessary to further validate their application within the context of Chinese culture.

## 7 Research prospects

(1) Broader Sampling: Future studies should consider selecting samples from diverse regions and various types of universities to conduct more comprehensive and in-depth research, aiming to achieve more generalizable results.
(2) Diverse Methodologies: Employing a range of approaches and methods can enhance the research. Data collection could be improved through techniques such as in-depth interviews, experiments, or quasi-experimental methods to acquire more authentic and reliable data. Additionally, incorporating case studies and other research methods could provide further insights.
(3) Extended Analysis: This study focused on the relationship between emotional labor and job burnout, and the moderating role of emotional intelligence in this relationship. Future research should investigate the specific role of emotional labor in the interplay between emotional intelligence and job burnout.

## Data Availability

The original contributions presented in the study are included in the article/supplementary material, further inquiries can be directed to the corresponding author.
